# The global compendium of *Aedes japonicus:* An Underappreciated Arbovirus Vector of growing concerns

**DOI:** 10.1038/s41597-026-07481-z

**Published:** 2026-06-16

**Authors:** Outammassine Abdelkrim, Daoudi Mohamed, Elmoutamanni Achraf, Calzolari Mattia, Moreno Ana, Davide Lelli, Francesco Defilippo, Mustapha Debboun, Boumezzough Ali, Zouhair Said, Ndao Momar

**Affiliations:** 1 Laboratoire de Lutte contre les Maladies Infectieuses, Faculté de Médecine et de Pharmacie, Université Cadi Ayyad, Marrakech, 40000 Morocco; 2 Infectious Diseases and Immunity in Global Health Program, Research Institute of the McGill University Health Centre, Montreal, Canada; 3 Department of Microbiology and Immunology, McGill University, Montreal, Canada; 4 AgroBioSciences Department (AgBS), College of Agriculture and Environmental Science (CAES), Mohammed VI Polytechnic University, 43150 Benguerir, Morocco; 5 Istituto Zooprofilattico Sperimentale della Lombardia e dell’Emilia Romagna “Bruno Ubertini”, Brescia, 42124 Italy; 6 Baylor College of Medicine, 1 Baylor Plaza, Houston, TX 77030 USA; 7 Microbial Biotechnologies, Agrosciences and Environment Laboratory (BioMAgE), Faculty of Sciences Semlalia, Cadi Ayyad University, Marrakech, Morocco; 8 Laboratory of Bacteriology-Virology, Avicienne Hospital Military, Marrakech, Morocco; 9 Division of Experimental Medicine, McGill University, Montreal, Canada; 10 National Reference Centre for Parasitology, Research Institute of the McGill University Health Centre, Montreal, Canada

## Abstract

The Asian bush mosquito, *Aedes japonicus* (Theobald, 1901), is an invasive species and a competent vector for several arboviruses, including chikungunya virus, dengue virus, Japanese encephalitis virus, West Nile virus, and Zika virus. Field studies have also detected La Crosse virus in wild populations, further supporting its potential role in arbovirus transmission. To address the fragmented and incomplete state of knowledge regarding its spread, we have assembled a global dataset of documented presences from 1950 to 2025. This data descriptor presents a curated database of geolocated records, formatted as points, derived primarily from peer-reviewed literature and supplemented with validated national survey data and selectively integrated records from the Global Biodiversity Information Facility (GBIF) following rigorous quality control. We detail the methodology for data acquisition, coordinate assignment, and the rigorous validation steps applied. This first comprehensive repository specifically for *Ae. japonicus*, containing 4618 validated records, provides a critical resource for spatial mapping and risk assessment of this vector and its associated pathogens.

## Background & Summary

The relentless global expansion of invasive mosquito species, propelled by intensifying international trade, human mobility, and climate change, constitutes a significant and growing threat to public health. Their capacity to serve as vectors for debilitating arboviruses such as dengue, chikungunya, yellow fever, and Zika places a heavy burden on health systems worldwide^1–3^. While the spotlight has historically fallen on *Aedes aegypti* and *Ae. albopictus*, a broader assemblage of *Aedes* species is now demonstrating a concerning potential to facilitate and amplify disease transmission in new regions^4^. The global distribution of major arbovirus vectors has been systematically mapped in landmark studies, most notably the comprehensive compendium by Kraemer *et al*.^1^, which established the global distributions of *Aedes aegypti* and *Aedes albopictus* and provided a methodological framework for vector mapping at a global scale. Building upon this foundation, subsequent research has emphasized the need to extend similar standardized approaches to other emerging invasive *Aedes* species^1^. Among these emerging threats, *Aedes japonicus* (Theobald, 1901), the Asian bush or rock pool mosquito, stands out as a particularly adept invader with proven vector competence, yet it remains critically underappreciated in global health discussions^5^.

Native to temperate regions of Japan, Korea, Taiwan, and parts of China and Russia, *Ae. japonicus* has exhibited a remarkable capacity for transcontinental spread. It holds the distinction of being the third invasive *Aedes* species to establish itself in Europe and is ranked among the world’s “100 worst” invasive alien species^6–8^. Its invasive success is multifactorial, underpinned by a suite of adaptive traits: high ecological plasticity, allowing it to exploit a wide range of natural and artificial water containers; remarkable cold tolerance, enabling it to overwinter in temperate climates where other *Aedes* vectors might perish; and a demonstrated ability to outcompete and displace native mosquito species in newly invaded territories^9,10^. This displacement can reshape local mosquito communities, with potential implications for the transmission dynamics of both endemic and introduced pathogens.

The public health risk posed by *Ae. japonicus* is not merely theoretical. Laboratory vector competence assays and field evidence have confirmed its ability to transmit a spectrum of viruses of major medical importance. It is a competent laboratory vector for Japanese encephalitis virus (JEV), West Nile virus (WNV) and has also been shown to transmit chikungunya (CHIKV), dengue (DENV), and Zika (ZIKV) viruses under experimental conditions^11–13^. Its role in the transmission cycle of WNV in North America is particularly well-documented, marking it as a key bridge vector between avian reservoirs and human populations^5,14^. The recent experimental demonstration of its ability to transmit Usutu virus further broadens its potential disease portfolio^13^. *Aedes japonicus* has demonstrated laboratory competence for several arboviruses; however, its role in natural transmission cycles remains limited. Notably, field detections of La Crosse virus (LACV) in *Ae. japonicus* populations in the United States suggest a potential role in the ecology of this endemic arbovirus. While evidence for sustained transmission is still limited, these findings highlight the importance of continued surveillance and evaluation of the species’ vector competence in regions where LACV is endemic^15^.

Despite these clear risks, the global data on the distribution of *Ae. japonicus* are characterized by fragmentation and inaccessibility. Occurrence records are scattered across hundreds of individual studies in multiple languages, reported in heterogeneous formats (from precise GPS coordinates to vague locality names), and buried in non-digitized reports from local health departments. This data siloing presents a fundamental barrier to a synthetic understanding of the species’ distributional ecology. Without a unified, standardized, and georeferenced dataset, it is impossible to accurately model its ecological niche, forecast its future spread, or assess the compounded risk of arbovirus emergence in regions on the front lines of its invasion.

This manuscript directly addresses this critical data gap. We present the first comprehensive, global compendium of *Ae. japonicus* occurrences, constructed through a systematic and reproducible methodology. By integrating and standardizing disparate data sources into a single, publicly accessible resource, we provide an essential evidence base. This dataset empowers the scientific and public health communities to move from reactive case-studies to proactive, predictive analyses of this invasive vector, ultimately supporting the development of more effective surveillance and control strategies to mitigate its impact on global health.

## Methods

### Data collection

A comprehensive search for occurrence records was performed using the online databases PubMed, Web of Science, and Scopus. The search incorporated the terms “*Aedes japonicus*”, “*Ochlerotatus japonicus*”, and “*Hulecoeteomyia japonica*” and covered the period from 1950 to 2025. Publications in any language were considered, provided a title and abstract were available. The initial search in PubMed returned 2,122 items; from these, 1742 publications were identified as containing potentially usable spatial data.

In parallel, we evaluated the Global Biodiversity Information Facility (GBIF www.gbif.org) as a complementary source of occurrence data. Given the heterogeneous nature of GBIF records, we adopted a conservative integration strategy, retaining only subsets associated with expert-validated surveillance systems. Specifically, GBIF-derived records were filtered to include data from: AIMSurv (a harmonized pan-European surveillance framework with standardized protocols and morphological and molecular validation), Mosquito Alert (citizen science data subjected to triple-expert entomological validation via the Entolab platform), VectorNet (a coordinated European surveillance network with standardized georeferencing and validation procedures), as well as curated datasets from the Institute of Tropical Medicine Antwerp, the National Museum of Nature and Science, Japan, and the European Food Safety Authority. This selective integration ensured that all retained GBIF records met the same quality standards as literature-derived occurrences. We explicitly excluded iNaturalist due to documented high misidentification rates (40% for *Ae. japonicus*/*Ae*. *koreicus*)^16^. In addition to published literature, we obtained 17 occurrence records (0.48% of the final dataset) from national entomological surveys through direct correspondence with Mattia Calzolari of the Istituto Zooprofilattico Sperimentale della Lombardia e dell’Emilia Romagna, Italy. These unpublished records were validated using the same criteria as published data. A record was deemed ‘confirmed’ if the source explicitly reported the presence of *Ae. japonicus* (in any life stage) at a definable location and date, including observations of non-established populations. All data incorporated into the database were obtained from publicly accessible online sources or through direct expert collaboration. The database was temporally standardized to avoid duplicates by defining a single occurrence per unique location per calendar year. This two-step procedure involved disaggregating multi-year records into yearly entries, then aggregating all records sharing the same location and year.

### Geo-positioning

Site descriptions and locality names were extracted from each qualifying publication. Geographic coordinates were assigned using online tools including Google Maps, Google Earth, and Geonames. Records that could be resolved to a specific position within a 5 × 5 km grid cell were categorized as ‘point’ locations. Each location was associated with its corresponding national-level administrative boundary as defined by the FAO Global Administrative Unit Layer (GAUL) system.

### Database Management: Spatial and Temporal Standardization

To consolidate potential duplicates across both literature-derived and GBIF-derived records, point occurrences falling within the same 5 × 5 km grid cell were assigned a shared unique identifier. We defined a single occurrence for any unique location within a given calendar year. Records spanning multiple years were disaggregated into separate annual entries, while multiple records from the same location and year (whether originating from repeated field sampling, longitudinal surveillance, or overlapping sources (e.g., literature and GBIF)) were merged to avoid pseudoreplication. Such repeated records often reflect multiple observations of the same underlying population rather than independent occurrences. This standardization process eliminated 700 duplicate entries and ensured that the dataset represents presence per location per year, preventing sampling intensity from biasing distribution analyses. Polygon records exceeding 1 degree in size (approximately 111 km at the equator) were excluded from the final dataset (n = 27).

## Data Records

The full dataset is available for download as a single Excel file, containing two sheets: “Data,” which includes the georeferenced occurrence records, and “References,” which documents the published sources used to compile the data record^17^. Each entry corresponds to a unique occurrence record, characterized by the following fields:

VECTOR: Species (*Ae. japonicus*).

OCCURRENCE_ID: Unique identifier.

SOURCE_TYPE: Published, GBIF or unpublished source.

LOCATION_TYPE: Point.

X: Longitude of point or polygon centroid (WGS84).

Y: Latitude of point or polygon centroid (WGS84).

YEAR: Year of occurrence.

COUNTRY: Country name.

COUNTRY_ID: ISO alpha-3 code.

GAUL_AD0: Global Administrative Unit Layer code for country-level administrative boundaries (FAO GAUL system). Values correspond to national codes; −999 indicates no GAUL code assigned (typically for points not associated with GAUL polygons).”

STATUT: Establishment status of the population at the time of collection, derived from the source publication. Categories include: ‘Established Introduced’ (self-sustaining populations in introduced range with evidence of reproduction and overwintering), ‘Recently Introduced’ (newly detected populations where long-term establishment is not yet confirmed), and ‘Native’ (records from the species’ native range in East Asia, including Japan, Korea, Taiwan, and parts of eastern China and Russia).”

## Technical Validation

**Fig. 1:**
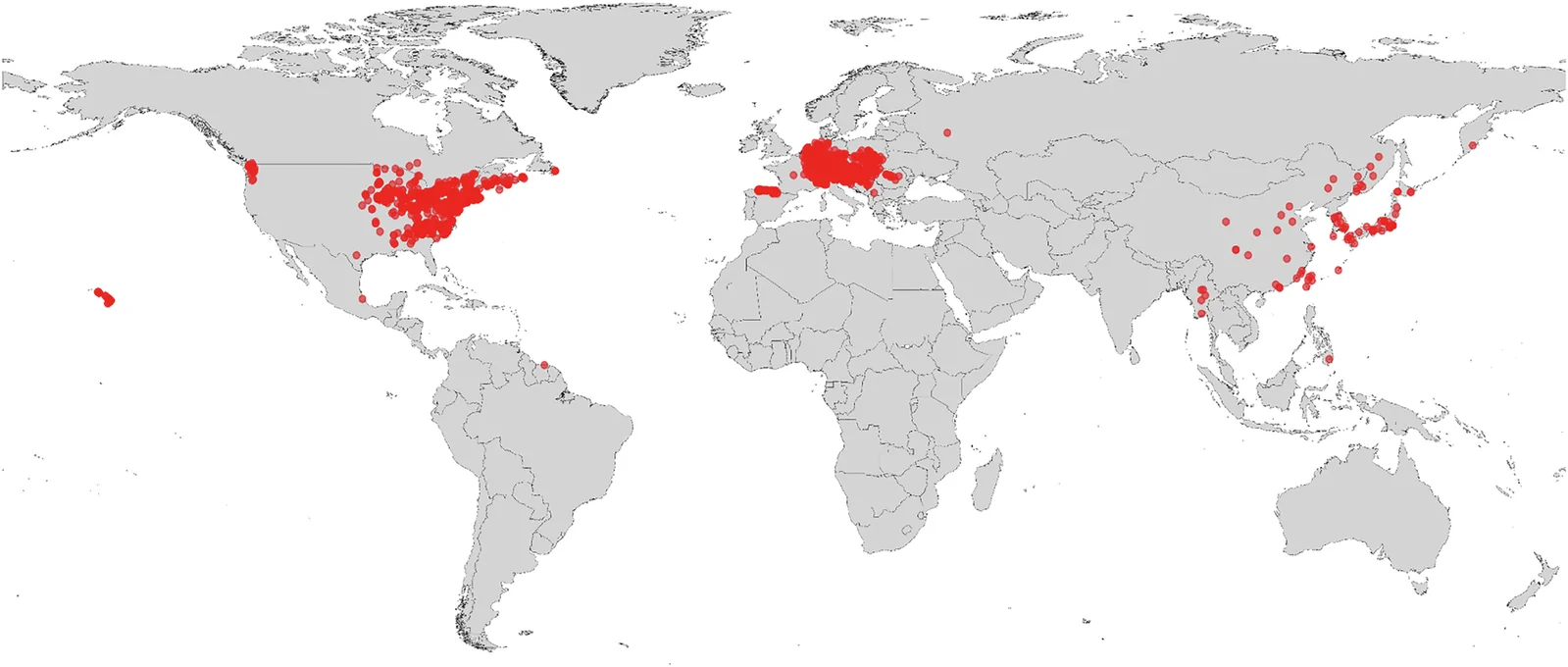
Global distribution of validated *Ae. japonicus* occurrence records (1950–2025). Georeferenced occurrence records compiled from published literature and official reports, representing the confirmed global distribution of *Ae. japonicus*. Points indicate validated records included in the curated dataset (n = 4618).

**Fig. 2:**
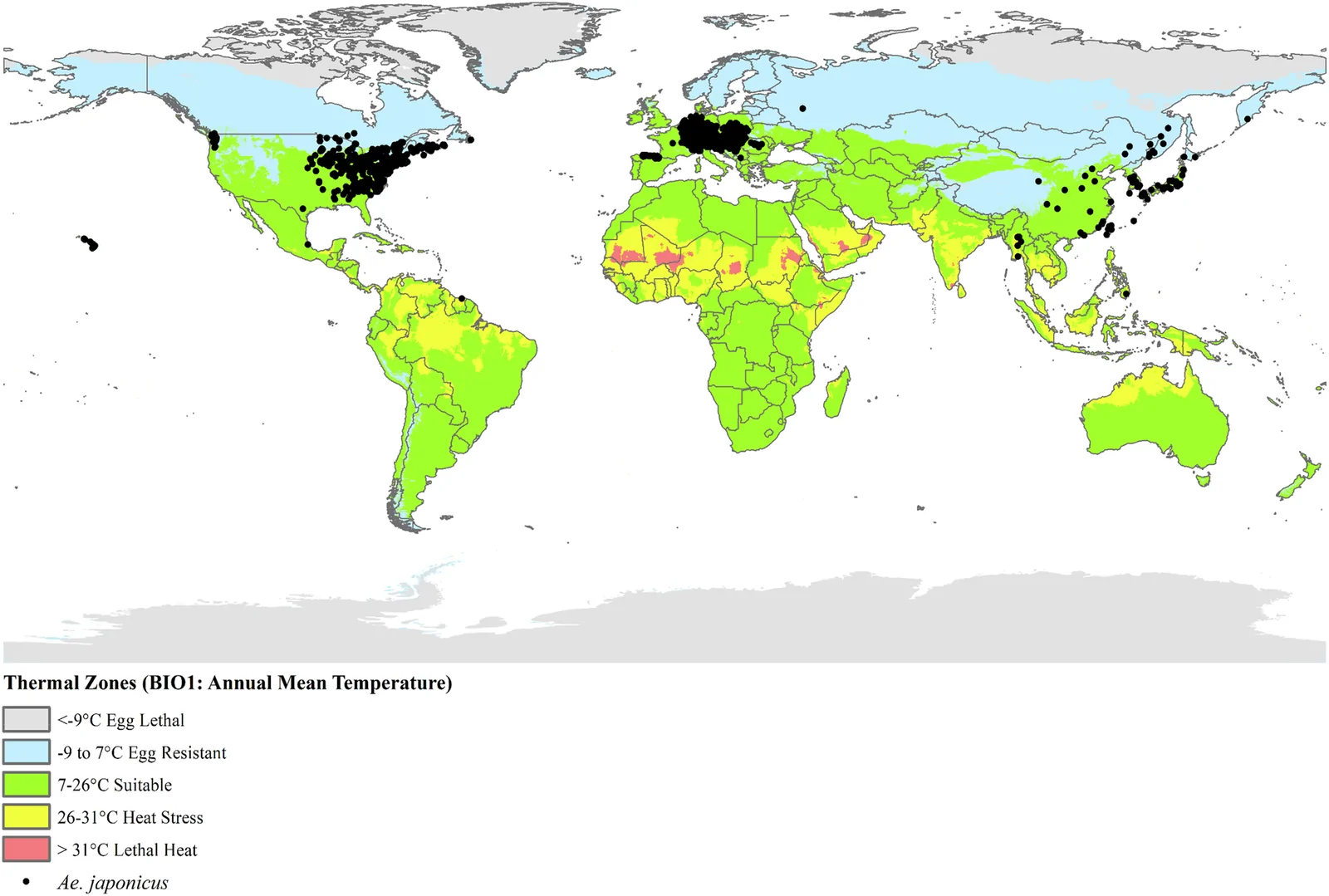
Global distribution of *Ae. japonicus* in relation to thermal suitability zones. World map showing occurrence records of *Ae. japonicus* overlaid on thermal zones derived from BIO1 (Annual Mean Temperature) climate data. Zones defined according to experimental thermal biology^20^: grey (<−9 °C, egg lethal), light blue (−9 to 7 °C, egg resistant/diapause capable), green (7–26 °C, thermally suitable for development), yellow (26–31 °C, heat stress zone), and red (>31 °C, lethal heat). Black dots indicate confirmed *Ae. japonicus* occurrence records (n = 4618).

**Fig. 3:**
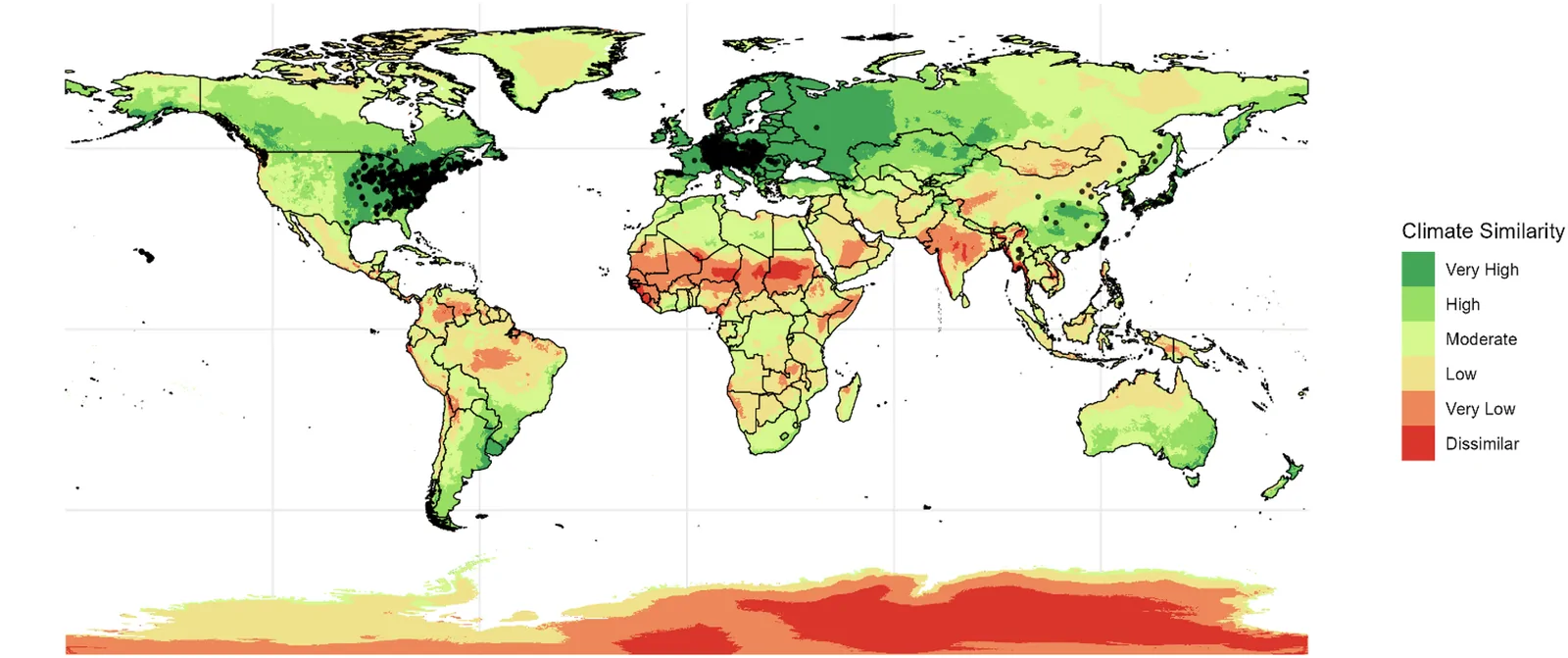
Climate similarity validation using Mahalanobis distance analysis. The map displays areas sharing similar climatic conditions to known *Ae. japonicus* occurrences, calculated from 19 wordclime bioclimatic variables. Climate similarity classes (Very High to Dissimilar) are based on percentile thresholds of Mahalanobis distance distribution. Black points show occurrence records, demonstrating that 98% of records fall within High or Very High similarity classes, confirming strong ecological coherence.

The dataset underwent a multi-step validation process to ensure spatial and ecological accuracy (Fig. 1):

1. Spatial Accuracy Verification: A global land cover raster was used to verify that all point locations fell on landmasses, excluding marine and aquatic environments^18^.
2. Climatic Plausibility Assessment: Following the methodological framework established by Brady *et al*.^19^ for validating occurrence records against climatic constraints, we assessed all *Ae. japonicus* records against species-specific thermal thresholds derived from experimental literature and ecological data. Based on thermal tolerance experiments and established physiological limits^20^, we evaluated occurrence locations using WorldClim bioclimatic variable BIO1 (Annual Mean Temperature) (Fig. 2). We applied a lower thermal limit of −9 °C, reflecting temperatures below which egg survival is lethal due to insufficient cold hardiness even with diapause^21^. We applied an upper thermal limit of 31 °C, corresponding to temperatures beyond which chronic heat stress significantly impairs larval development, adult survival, and reproductive success. The intermediate thermal zones were further categorized to reflect graded physiological responses: −9 to 7 °C represents the egg diapause-resistant zone where cold-tolerant eggs can overwinter; 7–26 °C represents the optimal suitable range for active development and population persistence; and 26–31 °C represents heat stress conditions where populations may persist but with reduced fitness. Of the 4,618 validated occurrence records, 4,353 (94.3%) fell within the suitable range (7–26 °C), 251 (5.4%) within the cold-resistant zone (−9 to 7 °C), 17 (0.4%) in the lethal cold zone (<−9 °C), and 5 (0.1%) in the heat stress zone (26–31 °C). No records (0%) exceeded the upper lethal threshold (>31 °C). The predominance of records within suitable and cold-resistant zones supports the ecological validity of the compiled distribution and indicates full climatic plausibility.
3. To further validate the ecological coherence of the occurrence distribution, we performed a Mahalanobis distance analysis using 19 bioclimatic variables from WorldClim. This multivariate statistical approach identifies areas sharing similar climate conditions to the known occurrence points, serving as an independent validation of the species’ climatic niche. The analysis revealed that 98% of our occurrence records fall within “High” or “Very High” climate similarity classes (Mahalanobis distance <25th percentile), confirming strong ecological consistency across the global distribution (Fig. 3). This approach follows established methodologies for validating species occurrence data against background climate conditions^22,23^. We acknowledge that the Mahalanobis distance analysis, like all niche modeling approaches, is susceptible to the same sampling biases discussed in the Usage Notes. The climate similarity surface reflects the environmental space occupied by our occurrence records, which are themselves subject to geographical sampling bias (e.g., oversampling in Europe and North America). Consequently, areas classified as “Very High” climate similarity may partly reflect the environmental conditions of well-sampled regions rather than the full fundamental niche of the species. However, the inclusion of records from the native range (East Asia) and under-sampled regions helps mitigate this bias. The finding that 98% of records fall within “High” or “Very High” similarity classes, therefore, primarily validates internal consistency, i.e., that most records occur in environments similar to one another, rather than providing an unbiased assessment of global niche suitability. Users should interpret the climate similarity map as an exploratory validation tool rather than a definitive niche model, and we recommend that formal species distribution modeling incorporate explicit bias correction methods as outlined in the Usage Notes.

The final curated dataset comprises 4618 georeferenced occurrence records spanning multiple continents and climatic regions. The global distribution of these records is shown in Fig. 1.

## Usage Notes

This consolidated dataset provides an unprecedented resource for analyzing the spatial and temporal dynamics of *Ae. japonicus* on a global scale. The primary utility of this database lies in its application across several critical domains of public health entomology and biogeography:

1. Tracking Invasion History and Pathways: The temporal depth of the dataset (1950–2025) allows for a retrospective analysis of the species’ invasion chronology. Researchers can track the progression from its native range in East Asia to its established populations in North America and Europe, and more recent incursions into new territories. By correlating first-detection records with global shipping routes, major road networks, and air traffic data, it is possible to model the most probable introduction pathways. This historical reconstruction is crucial for identifying key ports, airports, and trade hubs that act as gateways, enabling targeted surveillance and pre-emptive control measures in similar, yet uninvaded, regions.
2. Refining Ecological Niche and Species Distribution Models (SDMs): This dataset serves as the fundamental occurrence layer for robust ecological niche modeling. The inclusion of points records, all linked to environmental covariates, allows for the prediction of the species’ fundamental and realized niche. These models can identify regions with suitable climatic conditions (e.g., temperature, precipitation, humidity) that are currently uncolonized but at high risk of future establishment. This is particularly important for proactive public health planning in regions like South America, Africa, and Australasia, where suitable conditions exist but the species is not yet widely reported.
  Furthermore, the dataset can be used to project future distributions under various climate change scenarios, illustrating how the potential range of *Ae. japonicus* might shift or expand in coming decades.
3. Assessing Arboviral Transmission Risk: The vector competence of *Ae. japonicus* for viruses like JEV, WNV, and ZIKV elevates its public health significance beyond a mere nuisance. This dataset can be layered with data on human population density, the distribution of other primary vectors (like *Ae. aegypti*), and the geographic range of arboviruses. This integrated approach allows for the identification of “hotspot” regions where the confluence of a competent vector, a susceptible human population, and a circulating pathogen creates a high risk for disease emergence or amplification. For instance, in regions of Europe and North America where WNV is endemic, the expanding range of *Ae. japonicus* could alter local transmission dynamics, potentially acting as a bridge vector between avian reservoirs and human populations.
4. Informing Vector Surveillance and Control Strategies: Public health agencies often operate with limited resources. This dataset provides evidence-based guidance for prioritizing surveillance efforts. Areas predicted to be highly suitable by models, or those adjacent to known infestation zones along major transport corridors, can be flagged for enhanced monitoring. The data on establishment status (“established” vs. “transient”) is critical for response planning. The detection of a transient population may call for localized eradication campaigns, while data on established populations necessitates long-term, integrated vector management strategies focused on larval source reduction and community engagement.
5. Understanding Ecological Impacts and Community Interactions: As an invasive species, *Ae. japonicus* can displace native mosquito species and alter local ecosystem dynamics. This dataset provides a baseline for studying these ecological interactions. By comparing the distribution of *Ae. japonicus* with that of native and other invasive mosquitoes over time, researchers can investigate patterns of competitive exclusion or co-occurrence, which can have downstream effects on local disease transmission cycles.

### Critical Consideration on Sampling Bias

A paramount consideration for users is the inherent geographical sampling bias within the dataset. The density of records is not a true reflection of global mosquito abundance but is heavily influenced by disparities in research funding, public health infrastructure, and surveillance intensity. Europe and North America are disproportionately represented due to well-funded invasive species monitoring programs, while large parts of Africa, Asia, and South America are likely under-sampled. This bias can severely distort model predictions if not properly accounted for.

To mitigate this, analysts must employ techniques designed for presence-only data with sampling bias. This includes:

- Using Target-Group Backgrounds: Selecting background or pseudo-absence points from a bias layer representing sampling effort (e.g., all mosquito surveillance locations, not just random points across the landscape).
- Environmental Filtering: Ensuring that background points are drawn from environments similar to those where surveillance actually occurs.
- Explicit Bias Correction: Methods such as the Maxent model’s “bias file” can be used to down-weight the influence of oversampled regions.

Failure to account for this bias will result in models that reflect the geography of scientific collection rather than the true ecological preferences of *Ae. japonicus*, thereby limiting the predictive power and practical utility of the results.

### Critical Considerations on Isolated Detections

Isolated detections of *Ae. japonicus* in climatically suitable but historically underreported regions should be interpreted with caution. These records, supported by published sources and falling within the species’ documented climatic tolerance, were retained in the dataset because they represent confirmed occurrences. In some cases, such detections may reflect recent introductions via international trade and transport rather than long-established populations. While they provide valuable insight into invasion pathways and potential range expansion, they may not indicate stable establishment. Consequently, these observations should be interpreted as early indicators of spread when assessing long-term distribution and ecological suitability. Interception records and port-of-entry detections, in contrast, were excluded from the dataset because they do not represent confirmed occurrences in the environment.

## Data Availability

All The curated global occurrence dataset of *Aedes japonicus* (1950–2025), comprising 4618 validated records, has been deposited in the Figshare repository and is available at https://doi.org/10.6084/m9.figshare.30589469. All data were compiled from published literature, GBIF and their reuse complies with the terms and conditions of the original data providers.

## Code availability

No custom code was generated or used during this study. All analyses were performed using standard functions available in R version 4.3.1 and Microsoft Excel.
